# *Listeria monocytogenes *virulence factor Listeriolysin O favors bacterial growth in co-culture with the ciliate *Tetrahymena pyriformis*, causes protozoan encystment and promotes bacterial survival inside cysts

**DOI:** 10.1186/1471-2180-10-26

**Published:** 2010-01-28

**Authors:** Valentina I Pushkareva, Svetlana A Ermolaeva

**Affiliations:** 1 Gamaleya Research Institute of Epidemiology and Microbiology, Russian Academy of Medical Sciences, Moscow, Russia

## Abstract

**Background:**

The gram-positive pathogenic bacterium *Listeria monocytogenes *is widely spread in the nature. *L. monocytogenes *was reported to be isolated from soil, water, sewage and sludge. Listeriolysin O (LLO) is a *L. monocytogenes *major virulence factor. In the course of infection in mammals, LLO is required for intracellular survival and apoptosis induction in lymphocytes. In this study, we explored the potential of LLO to promote interactions between *L. monocytogenes *and the ubiquitous inhabitant of natural ecosystems bacteriovorous free-living ciliate *Tetrahymena pyriformis*.

**Results:**

Wild type *L. monocytogenes *reduced *T. pyriformis *trophozoite counts and stimulated encystment. The effects were observed starting from 48 h of co-incubation. On the day 14, trophozoites were eliminated from the co-culture while about 5 × 10^4 ^cells/ml remained in the axenic *T. pyriformis *culture. The deficient in the LLO-encoding *hly *gene *L. monocytogenes *strain failed to cause mortality among protozoa and to trigger protozoan encystment. Replenishment of the *hly *gene in the mutant strain restored toxicity towards protozoa and induction of protozoan encystment. The saprophytic non-haemolytic species *L. innocua *transformed with the LLO-expressing plasmid caused extensive mortality and encystment in ciliates. During the first week of co-incubation, LLO-producing *L. monocytogenes *demonstrated higher growth rates in association with *T. pyriformis *than the LLO-deficient isogenic strain. At latter stages of co-incubation bacterial counts were similar for both strains. *T. pyriformis *cysts infected with wild type *L. monocytogenes *caused listerial infection in guinea pigs upon ocular and oral inoculation. The infection was proved by bacterial plating from the internal organs.

**Conclusions:**

The *L. monocytogenes *virulence factor LLO promotes bacterial survival and growth in the presence of bacteriovorous ciliate *T. pyriformis*. LLO is responsible for *L. monocytogenes *toxicity for protozoa and induction of protozoan encystment. *L. monocytogenes *entrapped in cysts remained viable and virulent. In whole, LLO activity seems to support bacterial survival in the natural habitat outside of a host.

## Background

The gram-positive pathogenic bacterium *Listeria monocytogenes *is a causative agent of listeriosis, a food-borne disease associated with such severe manifestations as meningitis, meningoencephalitis and miscarriages in pregnant women. High mortality rates make listeriosis one of the most important issues among food-borne infections (for a review see [1,2]).

*L. monocytogenes *is found widely both in rural and urban environment. The pathogen isolation from soil, water, wildlife feeding grounds and plants has been reported [3-5]. Frequent isolation of *L. monocytogenes *from sewage and sludge has been also demonstrated [6]. Being ubiquitously distributed in the environment, *L. monocytogenes *may be involved in the interactions with free-living protozoa, a common representative of natural ecosystems.

It has been shown that *L. monocytogenes *is resistant to digestion by free-living amoebae and ciliates [7-10]. However, the mode of *L. monocytogenes *interactions with unicellular eukaryotes is less clear compared to its interactions with mammalian cells [1,11,12]. The cholesterol-dependent pore-forming haemolysin listeriolysin O (LLO) plays a major role in *L. monocytogenes *virulence for mammals (for a review see [13,14]. LLO is required for the mammalian host phagosome disruption and bacterial escape into the cytoplasm where *L. monocytogenes *multiplies [15]. In contrast *L. monocytogenes *lacking the LLO-encoding *hly *gene are not capable of proliferating in mammalian cells and hence are avirulent in murine model [16]. Besides its role in pathogen's intracellular replication, LLO can cause apoptosis in dendritic cells and lymphocytes during first days of infection in mice [17,18].

LLO expression is driven by the transcriptional regulator PrfA [2]. PrfA activity is lowest in rich medium such as Brain Heart Infusion at room temperature and increases with temperature or upon a shift into minimal medium. Mutations that lock PrfA in constitutively active conformation (PrfA*) cause LLO hyperexpression [19].

LLO is thought to be involved in the interactions between *L. monocytogenes *and protozoa as LLO-dependent release from digestive vacuole was observed in the amoeba *Acanthamoeba castellanii *[8]. However, the function of LLO in the interactions of *L. monocytogenes *with bacteriovorous protozoa is not fully understood. In this study, we examined the involvement of LLO in the interactions of *L. monocytogenes *and the ciliate *Tetrahymena pyriformis*.

The ciliates are common in the environment where *L. monocytogenes *encounters including soil, natural and anthropogenic water sources, sewage and sludge [20,21]. The majority of ciliates are bacteriovorous. Like other ciliates, *T. pyriformis *ingests food particles via the oral zone called a cytostome followed by formation of a food vacuole [22]. The vacuole circulates through the cytoplasm until the food is digested. *T. pyriformis *can undergo encystment, a protozoan response to adverse conditions and culture aging [21]. Encystment is accompanied by formation of resting non-feeding particles, cysts, which possess a protecting cell wall that preserves the cytoplasm [21]. *T. pyriformis *produces cysts at food deficiency, temperature changes, adverse pH and osmotic pressure [23]. The process of encystment is reversible as trophozoites can recover from cysts in favourable conditions.

We found that LLO production favours *L. monocytogenes *survival in association with *T. pyriformis*. Moreover, we have shown that *T. pyriformis *encystment is accelerated in co-culture with *L. monocytogenes *owing to LLO. In addition bacteria entrapped in cysts maintained viability and are capable of inducing infection in guinea pigs.

## Results

### A microscopic study of interactions between *L. monocytogenes *and *T. pyriformis*

**Figure 1:**
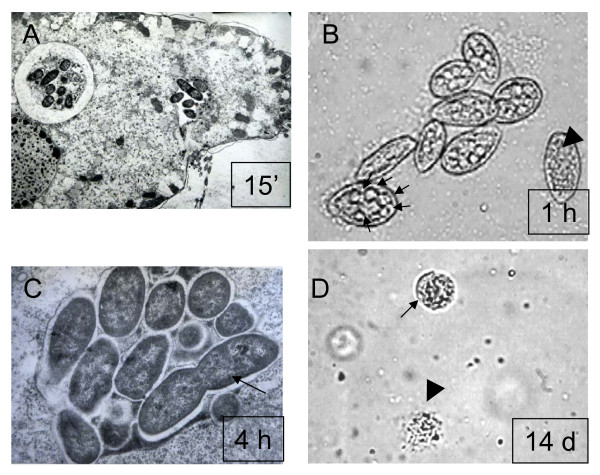
**A microscopic study of interactions between *L. monocytogenes *and *T. pyriformis***. A. Bacterial uptake by *T. pyriformis *in 15 minutes after the microorganisms were mixed. B. *T. pyriformis *cells in 1 h after the microorganisms were mixed. Multiple phagosomes within one cell are shown with arrows. *T. pyriformis *cell without phagosomes is shown with an arrowhead. C. Intraphagosomal bacteria. Dividing bacterium is shown with an arrow. D. Cysts (an arrow) and cell remnants (an arrowhead) after two weeks of incubation. The images were captured with transmission electron (A, C), or light (B, D) microscopy at magnification of 10 000 (A), 100 (B, D), and 25 000 (C).

The interactions between *L. monocytogenes *and *T. pyriformis *was studied by mixing *T. pyriformis *trophozoites with exponential culture of the wild type *L. monocytogenes *strain EGDe with MOI 1000:1 (bacteria/protozoa) in the LB broth and incubating at 28°C for up to 14 days. Active bacterial phagocytosis by protozoa was observed as soon as in 15 minutes after mixing (Figure 1A). In 1 h after bacterial addition, multiple vacuoles were observed inside the *T. pyriformis *cells (Figure 1B). Totally, 440 phagosomes were observed per 70 studied protozoan cells (Table 1). Each phagosome included from 5 to 15 bacteria as electron microscopy revealed (see Figure 1A and data not shown). Therefore, about 6,3 ± 3,1% of added bacteria were located intracellularly in 1 h after culture mixing. Undamaged bacterial cells were observed within phagosomes after 4 h, and some bacteria were dividing (Figure 1C).* T. pyriformis *cysts were observed together with trophozoites at later stages of incubation, and only cysts and cell remnants were revealed in the culture after 14 days (Figure 1D).

**Table 1:** Count of phagosomes formed by trophozoites in 1 h after addition of bacteria

Number of phagosomes per protozoan	0	5	6	7	8	9	10
Number of observed protozoa	5	14	18	16	7	6	4

### *L. monocytogenes *impairs growth of *T. pyriformis *and accelerates protozoan encystment

**Figure 2:**
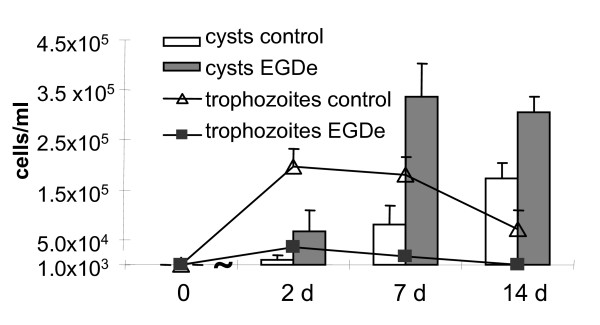
**Changes in the *T. pyriformis *population in the presence or absence of *L. monocytogenes***. Trophozoite concentrations are shown by polylines; cyst concentrations are shown by bars. Protozoa were grown alone (white) or in co-culture with the *L. monocytogenes *strain EGDe (solid). The mean values ± SD from three experiments made in triplicate are shown.

The growth of *T. pyriformis *infected by the wild type *L. monocytogenes *strain EGDe was significantly impaired compared to the control culture of protozoa grown alone under the same conditions (Figure 2). Cyst and trophozoites counts performed over the time from the same culture revealed about six-fold and ten-fold *L. monocytogenes*-associated reduction in the number of trophozoites on day 2 and day 7. On day 14 the number of trophozoites in the co-culture decreased below the detection limit, 10^3 ^cells/ml, (see Materials and Methods) while about 5 × 10^4 ^cells/ml remained in the control axenic culture of protozoa. Both cell death and cyst formation were responsible for disappearance of infected trophozoites (Figure 1D and Figure 2).

In the control culture, cysts were observed together with trophozoites starting from day 2; by the end of week 2, cyst concentration exceeded that of vegetative cells (Figure 2). Cyst formation might be due to depletion of nutrients exhausting by dividing protozoa. The presence of *L. monocytogenes *significantly accelerated protozoan encystment. Thus, on day 7 a four-fold increase in cyst concentration compared to the control culture was observed (p < 0.05). By the end of week 2 at least twice as more cysts compared to control and no vegetative cells were revealed in association with *L. monocytogenes*.

**Figure 3:**
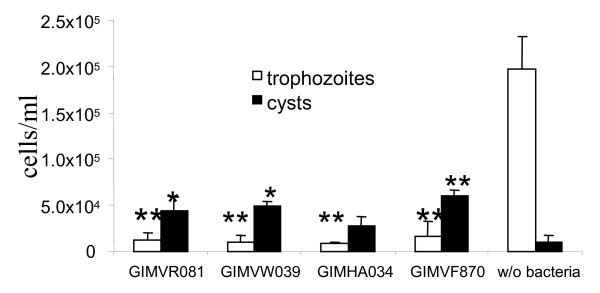
**The effect of various *L. monocytogenes *strains on the *T. pyriformis *population**. *T. pyriformis *trophozoite (while columns) and cyst (black columns) concentrations are shown on day 7 of co-cultivation with various *L. monocytogenes *strains designated on the figure, or without bacteria (w/o bacteria). The mean values ± SE from two experiments made in triplicate are shown.* p < 0,05; **p < 0,005.

To examine whether the observed effects are characteristic for wild type *L. monocytogenes *and were not associated with specific toxicity of the EGDe strain, four additional *L. monocytogenes *strains were tested. The previously described wild type strains VIMVR081, VIMVW039, VIMHA034, and VIMVF870 isolated from a wild rodent, environment, human, and food, respectively, were used [5]. All *L. monocytogenes *strains tested reduced trophozoite concentrations on day 7 (Figure 3). In contrast, the number of cysts increased in the co-culture. Thus, wild type *L. monocytogenes *caused mortality and induced encystment in *T. pyriformis*.

### LLO absence diminishes *L. monocytogenes *cytotoxic effect on *T. pyriformis *and prevents induction of protozoan encystment

**Figure 4:**
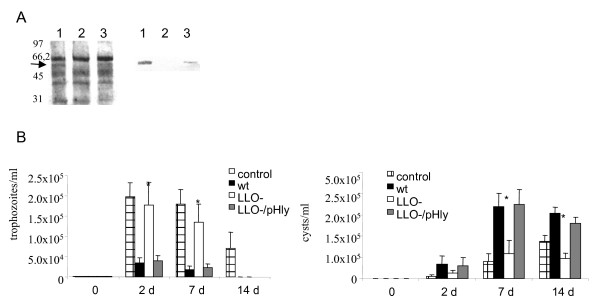
**Changes in the *T. pyriformis *population in the co-culture with *L. monocytogenes *in dependence on *L. monocytogenes *LLO production**. A. Detection of LLO in the culture supernatant of *L. monocytogenes *grown in the LB broth at 28°C. Proteins from 0,5 ml were loaded in each lane. On the left, secreted proteins are separated onto 10 % SDS-PAGE gel and visualized by staining with Coomassie Brilliant Blue R-250; on the right, Western blot analysis of secreted proteins with LLO-specific antiserum; 1 - wild type EGDe strain; 2 - EGDeΔ*hly *strain carrying the vector pTRKL2; 3 - EGDeΔ*hly *strain carrying the plasmid pHly. Numbers show molecular weight standard positions. The arrow shows a LLO position (MW_LLO _58 kDa). B. Trophozoite (left) and cyst (right) concentrations related to LLO production: designations for columns are shown on the figure. The mean values ± SE from two experiments made in triplicate are shown. * p < 0,05.

Using LLO specific antibodies, we observed that LLO expression was low but detectable in the conditions used (the LB broth at 28°C) (Figure 4A). Therefore, these conditions permitted us to examine a LLO contribution into the interactions between *L. monocytogenes *and *T. pyriformis*.

To investigate the importance of LLO for protozoan survival in co-culture with *L. monocytogenes*, the changes in *T. pyriformis *concentration were examined in the presence of the LLO deficient *L. monocytogenes *EGDeΔ*hly *strain with the *hly *gene removed by deletion. In contrast to the parental EGDe strain, EGDeΔ*hly *did not produce any decrease among alive trophozoites (Figure 4B) as well as no degraded cells (data not shown) were observed by day 7. Replenishment of the *hly *gene by introduction of a LLO-expressing pHly plasmid restored the cytotoxic phenotype of the EGDeΔ*hly *strain. However, by day 14 the concentration of trophozoites in co-culture with both *L. monocytogenes *strains could not be detected regardless on LLO production while trophozoites were present in the control axenic culture.

*L. monocytogenes *LLO deficiency decreased protozoan encystment pace in the bacterial presence. In fact, there was no significant difference in cyst concentration between *T. pyriformis *grown alone or in association with the Δ*hly *bacteria (Figure 4B). The functional *hly *gene located on the plasmid being introduced into the EGDeΔ*hly *strain restored bacterial ability to accelerate encystment. Therefore, toxic effects of wild type *L. monocytogenes *seemed to be due to LLO. Still, disappearance of trophozoites from the co-culture with the EGDeΔ*hly *bacteria suggested that other factors besides LLO might input into *L. monocytogenes *toxicity. *L. monocytogenes *phospholipases PlcA and PlcB, specific for phosphatidyl-inositol and phosphatidyl-choline [2], respectively, might be responsible for this effect.

### LLO-expressing *L. innocua *induces *T. pyriformis *mortality and encystment

**Figure 5:**
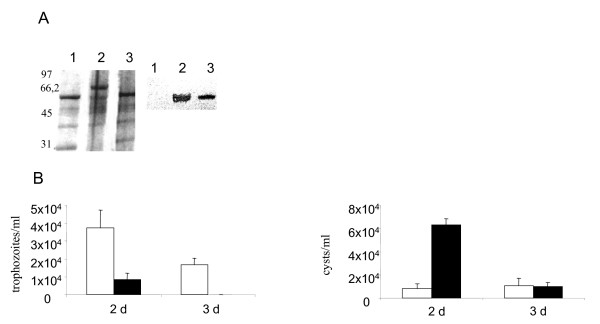
**Changes in the *T. pyriformis *population in co-culture with recombinant LLO-prodicing *L. innocua***. A. Detection of LLO in the culture supernatant of *L. innocua *and *L. monocytogenes*. On the left, secreted proteins are separated in the 10 % SDS-PAGE gel; on the right, Western blot analysis of secreted proteins with LLO-specific antiserum; 1 - wild type *L. innocua *NCTC11288 strain; 2 - *L. monocytogenes *NCTC 5105 strain; 3 - LLO-expressing *L. innocua *NCTC11288 (pHly/PrfA*) strain. Numbers show molecular weight standard positions. B. Trophozoite (left) and cyst (right) concentrations related to LLO production: while columns - *L. innocua *NCTC11288 strain; black columns - LLO-expressing *L. innocua *NCTC11288 (pHly/PrfA*) strain. Data represent mean ± SE of two experiments made in triplicate. * p < 0,05; **p < 0,005.

To confirm the role of LLO in *L. monocytogenes *toxicity, we checked an effect of LLO expression in non-haemolytic *L. innocua *on bacterial-protozoan interactions. *L. innocua *is a non-pathogenic species, which is closely related to *L. monocytogenes *[24]. Introduction of the pHly plasmid into the *L. innocua *strain NCTC 2188 did not result in detectable LLO production (data not shown). To improve LLO expression in *L. innocua*, we introduced the prfA* gene into the pHly plasmid. The prfA* gene encodes the PrfA* protein, which is a positive regulator of *hly *expression in *L. monocytogenes *[19]. *L. innocua *NCTC 2188 was transformed with the obtained plasmid designated as pHly/PrfA*. LLO production by the recombinant *L. innocua *strain carrying the pHly/PrfA* plasmid was evidenced by Western blotting (Figure 5A).

Introduction of the LLO-expressing plasmid produced a dramatic effect on the outcome of interactions between *L. innocua *and *T. pyriformis*. In 48 h in co-culture, trophozoite concentration diminished by a factor of four in the presence of recombinant *L. innocua *in comparison with a control, which was *T. pyriformis *co-cultivated with the parental *L. innocua *NCTC 2188 strain. Moreover, trophozoites totally disappeared in co-culture with LLO-expressing *L. innocua *after 72 h (Figure 5B). LLO-expressing *L. innocua *accelerated *T. pyriformis *encystment as it was previously observed with *L. monocytogenes*. At 48 h cyst concentration was about 7 fold higher in the presence of LLO-expressing *L. innocua *compared to the wild type strain. Interestingly, the cyst concentration diminished by a factor 5.6 between 48 h and 72 h, the effect was not observed in the presence of wild type *L. monocytogenes*. Obtained results supported a suggestion about a leading role of LLO in *L. monocytogenes *toxicity for protozoa.

### LLO supports *L. monocytogenes *survival in the presence of *T. pyriformis*

The next issue addressed was the *L. monocytogenes *survival in the presence of bacteriovorous *T. pyriformis *and its dependence on LLO production. Bacterial growth was measured in the sterile LB broth and in the presence of *T. pyriformis*.

**Figure 6:**
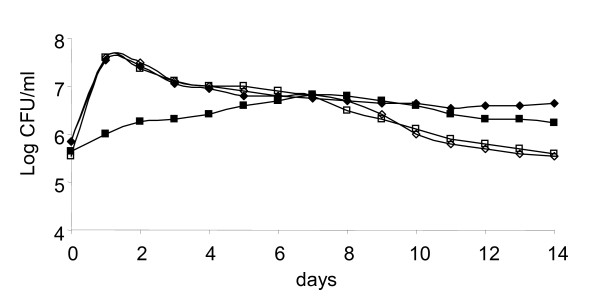
**Bacterial growth in dependence on the presence of *T. pyriformis *and LLO production**. White and solid symbols show *L. monocytogenes *grown alone and in the presence of *T. pyriformis*, respectively; triangles and squares are correspondent to the EGDe and EGDeΔhly strains, respectively. Bacterial concentrations were determined by plating of corresponding dilutions. A representative experiment from two replicates with similar results is shown.

Similar growth rates were observed for the wild type *L. monocytogenes *EGDe strain grown both alone or in association with *T. pyriformis *until end of week 1 (Figure 6). Later, bacterial population was stabilized in the association with *T. pyriformis *and higher bacterial concentrations were observed in the co-culture with *T. pyriformis *as compared with the control culture where *L. monocytogenes *grew alone. By the end of week 2 in the association with protozoa bacterial cell numbers exceeded the concentration of control bacteria by a factor of ten.

Deletion of the *hly *gene did not affect bacterial growth rates in the sterile LB broth. In contrast, *T. pyriformis *impaired the EGDe Δ*hly *growth especially during the first 5 days (Figure 6). By day 14, EGDeΔ*hly *concentration was higher in co-culture with protozoa than in the sterile LB broth. In whole, LLO deficiency deteriorated *L. monocytogenes *growth in the presence of a predator although the LLO-deficient bacteria appeared to be resistant to protozoan digestion that allowed maintaining a population at a relatively steady level.

### *L. monocytogenes *entrapped in cysts remains viable and virulent and causes infection in guinea pigs

**Figure 7:**
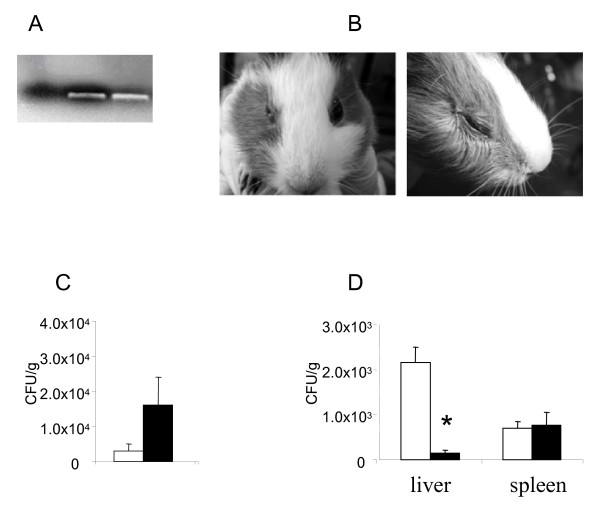
**Infection in guinea pigs caused by *L. monocytogenes*-infected *T. pyriformis *cysts**. A. qPCR products resolved on 2,5 % agarose. 1 - negative control, 2 - *L. monocytogenes *culture lysates, 3 - lysates of *T. pyriformis *cysts infected with *L. monocytogenes*. B. *L. monocytogenes *associated conjunctivitis. On the left, conjunctivitis of the right eye caused by *L. monocytogenes*, the left eye was not infected; on the right, conjunctivitis caused by *T. pyriformis *cysts carrying *L. monocytogenes*. C. *L. monocytogenes *isolated from faeces of animals infected orally with *L. monocytogenes *(while columns) or with *L. monocytogenes*-infected cysts (black columns). D - bacterial loads in the liver and the spleen of animals infected orally with *L. monocytogenes *(while columns) or with *L. monocytogenes*-infected cysts (black columns) after 72 h post-infection. Data were expressed as the mean ± SE for groups of three animals. X, only one animal gave feces after 24 h. * p < 0,05

The next question addressed was the fate of bacteria entrapped in the cysts. Bacterial presence in cysts, which were formed by day 7 in co-culture, was proposed on the base of positive PCR results (Figure 7A). However, no bacterial growth was observed when *L. monocytogenes *infected *T. pyriformis *cysts were directly plated on the LB agar. Bacteria in cysts might be dead or non-culturable.

To examine the viability and virulence potential of bacteria entrapped in cysts, we performed the infection of guinea pigs with *T. pyriformis *cysts. Stationary phase bacteria served a control. Bacterial loads were equalized using quantitative PCR (qPCR, Figure 7A). The inoculation of *L. monocytogenes*-infected cysts into guinea pig eyes induced acute conjunctivitis on days from 2 to 5 (Figure 7B). The eye injury ranged from moderate (closing of the palpebral fissure, epiphora, and photophobia) to severe (acute keratoconjunctivitis with edema and eyelid hyperaemia). Intact *T. pyriformis *cysts obtained by incubation of axenic trophozoites at +4°C overnight did not produce conjunctivitis.

To further examine the virulence potential of the bacteria clogged in *T. pyriformis *cysts, guinea pigs were orally infected with of cultured or entrapped in cysts *L. monocytogenes *with concentration 10^6 ^CFU/guinea pig as determined with qPCR. Bacterial counts in feces did not change significantly by day 2 being higher in cyst-infected animals (Figure 7). When all the infected animals were sacrificed on day 3 similar concentrations of *L. monocytogenes *were observed in spleen of the animals either infected by bacteria entrapped in cysts or grown in culture. The bacteria concentrations in the liver were about 20 times lower in the animals infected by bacteria entrapped in cysts compared to those obtained from culture.

## Discussion

Cytotoxicity of haemolytic *Listeria *spp. in ciliates and amoebae was originally demonstrated by Chau Ly and Müller [7]. They have shown that haemolytic *L. monocytogenes *and *L. seeligeri *induce lysis of *T. pyriformis *and *Acanthamoeba castellani *during 8-15 days while only few protozoa underwent lysis in the presence of non-haemolytic *L. innocua*.

Our results demonstrated that a *L. monocytogenes *mutant strain deficient in *L. monocytogenes *haemolysin, listeriolysin O (LLO) was incapable of impairing *T. pyriformis *growth compared to the isogenic wild type strain. A saprophytic species of *L. innocua *expressing LLO acquired toxicity in protozoa and caused their mortality and encystment. Thus, obtained results suggested that it is LLO that is responsible for *L. monocytogenes *cytotoxicity in protozoa.

Another observed LLO activity was stimulation of *T. pyriformis *encystment. Both cell death and encystment were responsible for decrease of trophozoite counts in the presence of *L. monocytogenes*. Here our results were in contradiction with previously published [7]. Although cited above authors found that *L. monocytogenes *accelerates encystment of *A. castellani*, they did not observe *T. pyriformis *encystment independently on bacterial presence [7]. This contradiction is related to the protozoan ability to encyst rather than LLO activity and might be due to different sources of a protozoan culture. Cyst formation by ciliates was described earlier [21] and cysts that we observed for the used *T. pyriformis *culture were similar to cysts depicted there (see Figure 1).

In contrast to wild type *L. monocytogenes*, LLO-expressing *L. innocua *caused a rapid decrease in counts not only trophozoites but as well cysts (see Figure 5). The constitutive LLO expression driven by PrfA* protein, which gene was inserted into the pHly/PrfA* plasmid, might be responsible for higher toxicity of *L. innocua *transformed with the plasmid. Wild type PrfA protein activity is regulated by co-factor binding, while the PrfA* protein is locked in the active conformation by a Gly145Ser substitution [19]. Obtained results suggested that PrfA activity and LLO expression by intracellular *L. monocytogenes *might be switch off after host cell encystment but this is not possible for PrfA* protein. Further studies with using L. monocytogenes prfA* [19] are needed to get evidences in support of this suggestion.

Another pathogenic bacterium, a common representative of natural ecosystems, *L. pneumophila *was demonstrated to be cytotoxic for amoeba and to kill *A. polyphaga *via induction of necrosis due to *Legionella pneumophila *pore-forming activity [25]. A similar mechanism might be responsible for the cytotoxic effect of LLO. LLO belongs to the family of cholesterol-dependent haemolysins, which includes streptolysin O and pneumolysin O [13,14]. Proteins of this family can form oligomeric rings that plunge into membrane and generate pores [26]. Therefore, LLO pore-forming activity might be responsible for *L. monocytogenes *cytoxicity in protozoa.

Our observations on the reduced growth of the *hly *gene deficient mutant in the co-culture with *T. pyriformis *compared to isogenic wild type bacteria are in line with a previous report that a *hly *gene deletion prevented *L. monocytogenes *from *A. castellanii *phagosome escaping [8]. Phagosome escaping is prerequisite for *L. monocytogenes *replication in mammalian but not insect cells [27]. It is not clear at present how the failure to escape the phagosome impairs intracellular growth in protozoan cells. However, the improved intracellular survival in synergy with rapid reduction of trophozoite concentration might be responsible for the advantages that LLO exerts on bacterial survival in the presence of actively grazing protozoa.

Considering the natural environment, LLO production seems to increase *L. monocytogenes *survival compared to non-haemolytic bacteria. Obtained results demonstrated higher counts for wild type *L. monocytogenes *than for the isogenic LLO deficient mutant during first days of co-cultivation supposing that wild type bacteria better survived upon initial interactions with the predator than non-haemolytic counterparts. Furthermore, prolonged bacterial survival might be supported by bacterial maintenance in protozoan cysts forming due to LLO activity. It is generally accepted that entrapped bacteria may benefit from the protective coat conferred by protozoan [28-30]. It has been demonstrated previously that encysted bacteria could survive sewage water treatment, which is fatal to free living bacteria [31]. Survival of human pathogens inside protozoan cysts was demonstrated previously for *Vibrio cholerae*, *L. pneumophila*, *Mycobacterium spp *and an avirulent strain of *Yersinia pestis *[32-34]. However, to our knowledge active stimulation of protozoan encystment by bacteria was demonstrated only in case of *L. monocytogenes *([7]; and this work). Maintenance of pathogenic bacteria within cysts not only protects them from unfavorable environmental conditions but as well can preserve at the first stages of interactions with the macroorganism. That might be an important mechanism for bacterial spreading in the natural ecosystems when cyst protection not only supports pathogen survival in the hostile environment but as well increases its chance to multiply upon host invasion.

Involvement of LLO in different aspects of interactions between *L. monocytogenes *and protozoa has a striking similarity with its multiples roles during infection in mammals. Phagosome membrane disruption is the major role for LLO in intracellular parasitism in mammalian cells [2,14]. However, LLO input in *L. monocytogenes *virulence is not limited to phagosome escaping: LLO generates a calcium flux into cells, promotes bacterial invasion in certain epithelial cells, and causes apoptosis in dendritic cells and T lymphocytes [13,17,18]. In protozoa, LLO is required for phagosome escaping likewise it takes place in mammalian cells [8]; it exerts a cytotoxic effect on protozoa ([7]; and this work); at last, our results suggested that LLO causes protozoan encystment. Possible parallels between LLO-mediated mechanisms causing apoptosis in immune cells and encystment in protozoa require a special investigation.

Despite the growing number of evidences that a prey-predator model describing interactions between protists and saprophytic bacteria, is not appropriate to explain the interactions of bacteriovorous protozoa and pathogenic bacteria, the mechanisms that permit pathogenic bacteria to avoid protozoan grazing are not clear. It was suggested that these mechanisms may involve at least in part the means that pathogens utilize to survive in higher eukaryotes [28-30,35]. Moreover, it was suggested that the resistance to digestion by bacteriovorous protozoa might be an evolutionary precursor of bacterial adaptation to intracellular survival in mammalian professional phagocytes such as macrophages. Our results support this hypothesis by demonstration of the role that the major virulence factor listeriolysin O (LLO) plays in interpopulation relationships of the pathogenic bacterium *L. monocytogenes *and the bacteriovorous ciliate *T. pyriformis*.

Discussing the input of LLO in interactions of *L. monocytogenes *with mammals and protozoa, it is necessary to take notice of LLO expression under different conditions. Expression of the PrfA protein, which is a master-regulator of virulence genes in *L. monocytogenes *[2], changes in a temperature-sensitive manner that results in very low expression of PrfA-controlled genes under environmental temperatures while their expression increases at the temperatures of mammalian body [36]. In contrast to other virulence factors, the LLO-encoding *hly *gene expression is regulated by both PrfA-dependent and PrfA-independent promoters [37]. Low LLO expression at environmental conditions driven by the PrfA-independent promoter and the low-active PrfA-dependent promoter is sufficient to provide *L. monocytogenes *with benefits in its interactions with other members of the natural ecosystems. Increasing LLO expression, e.g. via introduction of the PrfA* protein, which stimulates higher expression from the PrfA-dependent promoter, distorts the balance causing mortality not only among trophozoites but as well among cysts as we observed for *L. innocua *carrying pHly/PrfA* plasmid. Therefore, mutations resulting in increased LLO production might be detrimental for survival in the nature. It is interesting, that another *Listeria *virulent species, *L. ivanovii*, which is highly haemolytic and is not able to repress virulence factor production via a described PrfA-dependent mechanism [38], is much more rear isolated from environment than *L. monocytogenes *[39,40]. Thus, LLO expression might be beneficial under different conditions but it is required a tight regulation in dependence on external conditions.

## Conclusions

LLO exerts a toxic effect on protozoa and causes protozoan encystment. LLO production favors the *L. monocytogenes *growth in the presence of *T. pyriformis *and promotes bacterial survival inside protozoan cysts. Infected cysts cause specific bacterial infection in susceptible animals.

## Methods

### Microorganisms and growth conditions

Bacterial strains used in the study are listed in Table 2. The *Escherichia coli *JM109 strain was used as an intermediate host in cloning procedures. Bacteria were routinely cultured on LB agar plates at 28°C. For plasmid-carrying strains, the medium was supplemented with erythromycin (10 μg/ml and 300 μg/ml for *Listeria spp*. and *E. coli*, respectively). Axenic *T. pyriformis *from the Collection of the Gamaleya Institute was maintained on LB supplied by gentamycin 100 μg/ml, diflucan 100 μg/ml, cyfran 100 μg/ml at 28 °C. Antibiotics were removed 3 days before the onset of the experiment.

**Table 2:** Bacterial strains used in the study

Bacterium	Description	Reference
*L. monocytogenes*		
EGDe	Wild type, serovar 1/2a	[24]
EGDeΔ*hly*	The *hly *gene deletion	[19]
NCTC5105	The prfA* gene encoding constitutively active PrfA*, serovar 1/2a	[19]
VIMVR081	Wild type, wild rodent isolate, serovar 4b	[5]
VIMVW039	Wild type, environmental isolate, serovar 4b	[5]
VIMHA034	Wild type, clinical isolate, serovar 1/2a	[5]
VIMVF870	Wild type, food isolate, serovar 1/2a	[5]
*L. innocua*		
NCTC11288	Wild type, serovar 6a	[5]
*E. coli*		
JM109	*recA*1, *endA*1, *gyrA*96, *thi*, *hsdR*17, *supE*44, *relA*1, Δ(*lac-proAB*)/F' [*traD*36, *proAB*^+^, *lacI*^q^, *lacZ*ΔM15]	Fermentas (Lituania)

Three day old culture of *T. pyriformis *was diluted by fresh LB broth to a concentration of 10^3 ^cells/ml. Exponentially grown *L. monocytogenes *were introduced into protozoan culture with multiplicity 1000:1 (bacteria/protozoa). The co-culture was maintained at 28°C without agitation for 14 days. All experiments were performed in triplicate.

### Protozoan and bacterial growth quantification

The culture was shaken to keep the concentration of protozoa steady throughout the volume. Bacteria were counted by plating of serial dilutions of the culture on LB plates. 500 μl of suspension was mixed with equal volume of the Lili buffer (30 % acetic acid - 70 % ethanol) to fix ciliates. After that protozoan cells were counted using light microscopy.

### Plasmid construction

The DNA fragment carrying the *hly *gene including the promoters and the regulating element (PrfA box) was synthesized in PCR using hly1 and hly2 primers (hly1: 5' - AGAGCG**CTGCAG**GGTTTGTTGTGTC; hly2: 5' - TACGTT**CTGCAG**TAGAAACTATAGG; *PstI *recognition sites are highlighted in bold) and *L. monocytogenes *EGDe bacterial lysates obtained after bacterial cell treatment with lysozyme (2 mg/ml) at 37°C for 1 h and Proteinase K (100 μg/ml) at 56°C for 1 h followed by boiling for 10 min. The PCR product was inserted into the *PstI *restriction site of the shuttle vector pTRKL2 [41]. The insertion was sequenced to evidence the *hly *gene integrity. The obtained plasmid designated pHly was introduced into the EGDeΔ*hly *strain by electroporation as was described previously [42]. LLO expression was verified by Western blotting as described below.

To obtain a plasmid for LLO expression in *L. innocua*, the DNA fragment carrying the prfA* gene encoding the transcriptional regulator PrfA* was obtained in PCR using the lysate of the *L. monocytogenes *NCTC5105 cells (prfA* phenotype, [19]) and prf1 and prf2 primers (prf1: 5' - CCCAGTTCTTTCAGGTCCGGC; prf2: 5' - ACT CACGCAAATTCGGCATGC). PrfA regulator is necessary for the *hly *gene expression, the substitution Gly145Ser in the PrfA* protein results in constitutive PrfA protein activity and constitutive the *hly *gene expression [19]. The ends of the obtained PCR product were blunted with T4-polymerase. After that it was inserted into the SmaI restriction site on the pHly plasmid. The plasmid designated pHly/PrfA* was introduced into the *L. innocua *strain NCTC11288 by electroporation [42].

### SDS-PAGE and Western immunoblotting

*L. monocytogenes *and *L. innocua *strains were grown overnight on LB, supplemented with erythromycin 10 μg/ml when necessary, at 28°C. Secreted proteins present in 1.5 ml of cell free culture supernatant were precipitated on ice for 1 h with 10% trichloroacetic acid followed by centrifugation at 10 000 rpm for 30 min. The protein pellet was washed with 70% ethanol, resuspended in 1× Laemmli buffer and boiled for 5 min. Proteins were separated onto 10 % SDS-PAGE gels and visualized by staining with Coomassie Brilliant Blue R-250. For Western analysis proteins were transferred electrophoretically from SDS-PAGE gels onto the nitrocellulose membrane (Amersham) using a Mini-Protein Cuvette (Bio Rad). LLO was detected with polyclonal rabbit primary antibodies raised against the purified *L. monocytogenes *LLO [43], secondary horseradish peroxidase-conjgated goat anti-rabbit antibodies (Bio-Rad) and visualized with the TMB stabilized substrate (Promega).

### Sample preparation and PCR

The quantitative PCR (qPCR) was performed using bacterial lysates obtained after bacterial cell treatment with lysozyme (2 mg/ml) at 37°C for 1 h and Proteinase K (100 μg/ml) at 56°C for 1 h followed by boiling for 10 min. Bacteria-containing *T. pyriformis *cysts were subjected to ultrasound treatment for 1 min (4 cycles of 15 seconds at a maximal amplitude) and then to the same treatment as described above. The act1 and act2 primers and the TaqMan probe were specific for the *L. monocytogenes *chromosomal *actA *gene (act1: 5'-AAAGATGCGGGGAAATGGG; act2: 5'-TGGTGTCTCTGGCAAAGCA; TaqMan: act 5'-FAM-ATG-CTT-CGG-ACT-TCC-CGC-CAC-CAC-CTA-BHQ1). qPCR was carried out in a 25 μl reaction volume containing 1 μl of bacterial lysate, 5 pM of each primers, 2.5 pM of the TaqMan probe and 1 U of Taq-polymerase (qPCR degree, Syntol, Russia) with the ANK-16 amplification and detection system (Syntol, Russia). The following cycle profile was used: one cycle at 96°C for 2 min followed by 40 cycles at 96°C for 15 s, and 62°C for 30 s. DNA amounts were quantified by using a standard curve obtained with results of tenfold serial dilutions of lysates of 1 to 10^6 ^bacteria. All measurements were done in duplicate.

### Guinea pig infection

All experiments on animals were performed with the approval of the Animal Care and Use Committee of Gamaleya Institute of Epidemiology and Microbiology.

*T. pyriformis *and *L. monocytogene*s EGDe strain were co-cultured for 7 days in 100 ml LB broth at 28°C. On day 7 cyst concentration exceeded that of trophozoites. After that in the remaining vegetative cells the encystment was promoted by their incubation at +4°C overnight. This was followed by the removal of extracellular bacteria with gentamycin treatment (100 μg/ml) for 2 h at room temperature. Control bacteria were grown overnight on LB plates, suspended in 1 ml of PBS, diluted with PBS to a concentration of 10^9 ^CFU/ml and kept frozen in 10% glycerin.

Groups of three female 350 g guinea pigs were infected intraconjunctivally by applying a cotton wool tampon saturated with the *T. pyriformis *cyst water suspension at concentration 8.9 x10^4 ^cyst/ml, which contained 1 × 10^6 ^*L. monocytogenes *CFU/ml or with *L. monocytogenes *suspension at concentration 1 × 10^6 ^CFU/ml. Bacterial loads were equalized using qPCR as described above. Three guinea pigs were infected with 1 × 10^5 ^axenic *T. pyriformis *cysts as a control.

For oral inoculation, 1 ml of water suspension containing *L. monocytogenes *in concentration 1 × 10^6 ^CFU/ml (clogged in cysts or from the culture) was introduced to the back of oral cavity of three animals. The animals were not fed for 12 h before infection. The concentration of *L. monocytogenes *in faeces was determined daily by plating serial dilutions on the selective medium (PALCAM agar, HiMedia, India). On day 3 (72 h after infection) animals were anaesthetized by chloroform and sacrificed. The liver and the spleen were homogenized in PBS and serial dilutions of homogenate material were plated on LB agar.

### Microscopic studies

Transmission electron microscopic investigations were performed in general as described in [44]. In short, microorganisms were fixed with phosphate-buffered osmium tertraoxide according to [45], dehydrated in alcohols of increasing concentrations, and embedded in araldite M. Ultrathin sections were produced on an LKB-3 ultratome, and studied in a GEM 100B electron microscope. Up to six sections for one sample were studied. Light microscopic studies were performed with Olympus IX-71 microscope.

## Authors' contributions

VIP carried out experimental work with protozoa. SAE carried out the molecular genetic work and drafted the manuscript. Both authors read and approved the final manuscript.
